# Curing Kinetic Analysis of Acrylate Photopolymer for Additive Manufacturing by Photo-DSC

**DOI:** 10.3390/polym12051080

**Published:** 2020-05-09

**Authors:** Fengze Jiang, Dietmar Drummer

**Affiliations:** 1 Institute of Polymer Technology (LKT), Friedrich-Alexander-University Erlangen-Nuremberg, Am Weichselgarten 9, 91058 Erlangen, Germany; dietmar.drummer@fau.de; 2 Institute of Polymer Technology (LKT), Collaborative Research Center 814—Additive Manufacturing, Friedrich-Alexander- University Erlangen-Nuremberg, Am Weichselgarten 9, 91058 Erlangen, Germany

**Keywords:** UV curing, curing kinetic, photo-DSC, photopolymerization, additive manufacturing

## Abstract

In this research, the curing degree of an acrylate-based monomer using direct UV-assisted writing technology was characterized by differential photo calorimetry (Photo-DSC) to investigate the curing behavior. Triggered by the UV light, the duo function group monomer 1,6-Hexamethylene diacrylate (HDDA), photoinitiator 1173 and photoinhibitor exhibit a fast curing process. The exothermal photopolymerization reaction was performed in the isothermal mode in order to evaluate the different thermal effects that occurred during the photopolymerization process. The influences of both UV light intensity and exposure time were studied with single-factor analysis. The results obtained by photo-DSC also allow us to perform the kinetic study of the polymerization process: The results show that, for the reaction, the higher the UV intensity, the higher the curing degree together with faster curing speed. At the same time, the effect of the heat released during the exothermic reaction is negligible for the polymerization process. When increasing the exposure time, limited improvement of curing degree was shown, and the distribution is between 65–75%. The reaction enthalpy and related curing degree work as a function of time. The Avrami theory of phase change was introduced to describe the experimental data. The functions of a curing degree with light intensity and exposure time were achieved, respectively.

## 1. Introduction

Additive manufacturing (AM) has achieved significant progress in recent years and has been successfully applied in a variety of industries. Even though different additive manufacturing technologies have been introduced; however, the combination with several technologies has rarely discussed [1]. Among additive manufacturing, the direct writing (DW) process is one of the most potential AM processes for fabrication due to its compatibility with a variety of functional materials [2]. Ultraviolet (UV)-assisted direct writing, with the advantages of fused filament fabrication (FFF) and stereolithography (SLA), improves the printing speed and the parts’ quality based on its curing mechanism. The high-liquidity, high-quality interlayer bonding and fast curing properties are capable of generating complex three-dimensional structures and even self-supporting features with high resolution [3].

UV-curable resin plays a core role in this UV-assisted DW system. The need for functional materials that are compatible with the existing or newly invented technologies is one of the driving forces for researchers. The fast curing and energy-saving properties elicit enormous attention in industries. The resin formed via UV initiated polymerization is of great importance due to its wild applications in a variety of fields ranging from coatings [4], dental materials [5], sportswear [6] to aerospace industries [7]. The main advantages of UV-curable resin in comparison with the conventional solvent-based or heat-curing resin are the environmental benefits originating from the absence of volatile components in the system, the low energy consumption, together with the high speed and efficient curing at room temperature. The liquid resin transfers to a solid polymer with outstanding thermal and mechanical properties within a very short time through free radical polymerization. The reactive functional groups lead to the formation of polymeric crosslinked material by free radicals [8]. The fast crosslink reaction also improves the mechanical properties in a shorter processing time [9].

Photo-DSC has been widely utilized to elucidate the key cure-process parameters such as the extent of crosslinking, the curing degree, the polymerization rate and the kinetic order of reactions. During the polymerization, under UV irradiation, exothermic heat flow from the polymerization reaction induced by a UV lamp heats the sample. Calorimetric measurements were carried out by photo-DSC as a function of the intensity and exposure time of the UV lamp for the various samples [10,11].

There are several factors that affect the properties of the photopolymerization process. Assche investigated the effect of temperature and UV intensity on the result of total reaction heat and conversion. The obtained results demonstrated that higher temperature and light intensity lead to a higher reaction rate in a shorter polymerization time [12]. Hong discovered the curing kinetics of a cationic polymerization system and discovered that the higher isothermal temperature leads to a significantly higher reaction rate constant and the higher concentration of photoinitiator will reduce the activation energy for the reaction [10]. Belaid found that the composite composed of TPGDA and eutectic liquid crystal that the exothermic reaction was nearly independent of the dose and intensity of the UV radiation [11]. Michaud focused on photopolymerization kinetics and structure formation. An autocatalytic relation was used to describe the conversion state with Arrhenius and power law relationships for temperature and light intensity dependence [13]. Lin discovered the model of the analytic relationship between curing depth and crosslink time, removed the part geometry limitation and also investigated the effects of oxygen inhibition and viscosity [14,15].

The kinetics of the curing of resins, especially epoxy resins, have been extensively investigated during the past due to their vast industrial applications. The study of curing kinetics provides both the understanding of the process development and the improvement of the quality of the parts [16]. A comparison of the theoretical predictions with experimental data suggests that the Avrami theory that present phase change may model the cure kinetics of these material systems adequately [17].

However, most studies on UV curing materials in additive manufacturing are focusing on new processing technologies, processing parameters, parts properties and high-performance, multifunctional new materials’ development. The fundamental investigation of the material reaction and kinetics are comparatively limited and has not been systematically studied [17,18,19]. The commercial resin composition remains vague owing to its commercial concerns. Different monomers and photoinitiators might cause different curing reactions in coordination with their chemical structure and reaction mechanism. In order to comprehensively unearth the fundamental behavior of the parts, the material-based study is of an urgent need. The influences of UV intensity, temperature, isothermal curing and thermal curing on the overall polymerization kinetics and the resulting material properties for different material combinations is not fully quantified yet.

In this paper, the curing kinetics of the customized photosensitive resin was reported. The effects of UV light intensity and exposure time on the curing degree were investigated. Isothermal mode of photo-DSC was used to measure the heat flow and then integrate to the degree of curing of the resin. The degree of curing was carried out to evaluate the energy absorption. Additionally, the curing degree model as a function of time and intensity was established based on the Avrami equation.

## 2. Materials and Methods

### 2.1. Material

**Figure 1:**
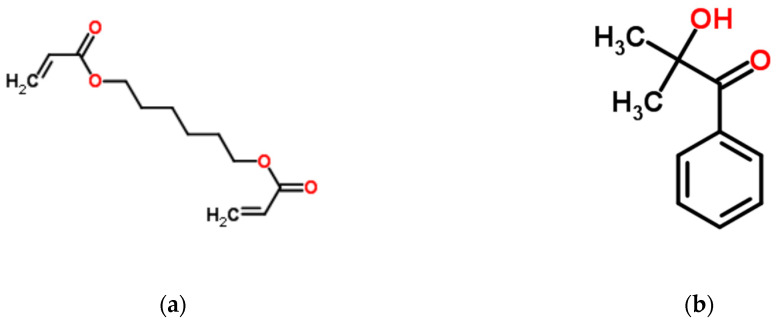
The chemical structures of resin components: (**a**) 1,6-hexamethylene diacrylate; (**b**) 2-hydroxy-2-methyl-1-phenylpropanone.

The monomer of the UV curing resin was 96 wt % 1,6-hexamethylene diacrylate (HDDA, Photomer 4017), with a density of 1.020 g/cm^3^ and the viscosity of 5–10 mPa·s at 25 °C. HDDA is a low viscosity diluent for UV radiation-curable lacquers and pigmented coatings, used in paper and board coatings, wood finishes vinyl flooring and flexible vinyl [20]. Inhibitor content has already been added into the monomer with a concentration between 100 and 500 ppm. The photoinitiator was prepared with the concentration at 4 wt % of the 2-hydroxy-2-methyl-1-phenylpropanone (Omnirad 1173) with a density of 1.08 g/cm^3^. The radical photoinitiator was chosen to ensure fast UV absorption and reaction. The most sensitive wavelength range for resin absorption is 244 and 330 nm. The monomer and photoinitiator were mixed with Thinky ARE-310 (Thinky INC., Laguna Hills, CA, USA) centrifugal mixer to keep a high mixing quality. Both monomer and photoinitiator were purchased from IGM Resins, the Netherlands. All materials were used as received. The chemical structures of the materials are shown in Figure 1.

### 2.2. Methods

The heat of the photopolymerization reaction was measured isothermally by using differential photo calorimetry DSC Q2000 (TA Instruments, New Castle, DE, USA). The UV light source Omnicure S2000 (Excelitas Technologies, Waltham, MA, USA) is connected to the DSC furnace with two optical fiber guides (focus on the sample and the reference pan). The light source is a high-pressure mercury short arc lamp that has a maximum intensity as high as 200 W/cm^2^. The emission spectrum radiated from this lamp was mostly between 320 and 500 nm.

**Figure 2:**
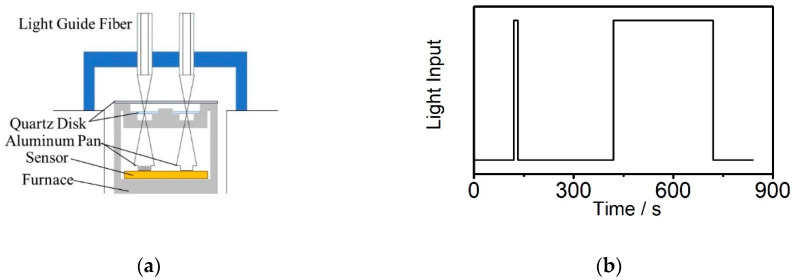
Photo-DSC structure and exposure program: (**a**) the cross-section structure of the photo-DSC furnace where (**b**) the curing program is composed of two parts: the preliminary exposure time and the complete cure time of the resin.

Shallow open aluminum pans were filled with a droplet of 1 mg of the mixed sample (resulting in a thickness of approx. 100 µm) and placed in a cell for UV experiments. The cell comprised lenses which focused the UV light onto the sample and reference pans are shown in Figure 2a. The sample-pan holders were sealed with a quartz window that let the UV light pass through. The sample space was kept in a nitrogen atmosphere. Each UV exposure sequence was repeated on the polymerized sample to acquire the baseline heat flow to be subtracted from the initial sequence. This baseline heat flow corresponds to the difference of UV absorption between the reference aluminum pan and the polymerized sample. Each experiment was repeated on three different samples.

The photo-DSC structure is shown in Figure 2a. The UV accessories are above the normal DSC furnace, the quartz disks isolate the light fiber and the furnace to avoid temperature effects. The UV light exposure is controlled by the shutter of the light source, the minimum time frame is 1 s. To make sure the resin is highly cured during the process, the measurement method was shown in Figure 2b. The first peak is the light exposure time which is one of the parameters settings, and the second peak is the extra exposure until no further heat release from the resin with certain light intensity. The isothermal holding step is stopped after no change of the heat flow can be detected [21]. The experiments were carried out in nitrogen at a flow rate of 50 cc/min and the DSC sampling interval was 0.2 s/pt.

**Figure 3:**
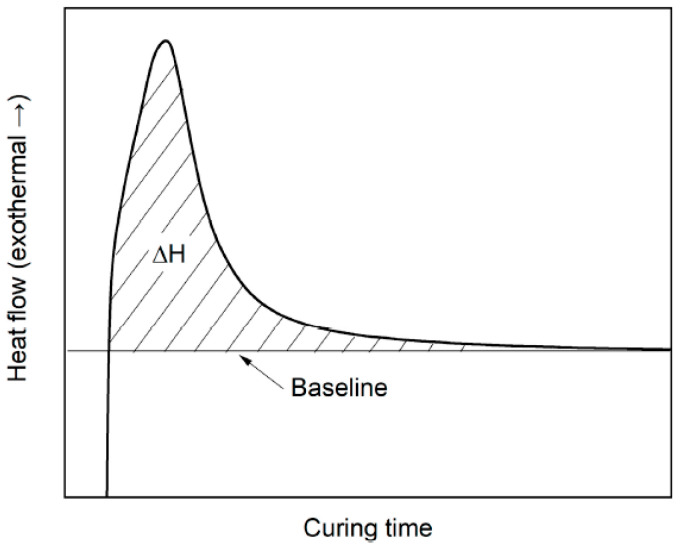
Enthalpy calculation schematic diagram [22].

When the resin is exposed under certain light intensity, the total heat flow of the resin will be fixed. In this case, when the resin is not cured, the theoretical enthalpy theoretically should be lower than the total fixed enthalpy. The recorded heat flow was used to track the reaction process of photopolymerization and to calculate the curing degree as well as the curing rate. The heat of reaction *H*_t_ was calculated by integrating the area under the exothermic peak, as shown in Figure 3.

The experimental extent of curing degree at time *t* was determined by relating the obtained heat of reaction at time *t* to the total measured heat of reaction after 5 min, Δ*H_total_*, after the heat flux remains stable. The curing degree α(*t*) was determined by relating the obtained reaction heat *H*_t_ at time *t* to the theoretical total reaction heat Δ*H_total_*. The *q*(*t*) was defined as the exothermal heat flux at time *t*. The curing degree *α*(*t*) can be defined as expressed in Equation (1). The baseline is a straight tangential line to the horizontal part of the isothermal DSC curve as shown in Figure 3. Each parameter was measured three times and the mean value was used for the calculation of the curing degree.

(1) $$\alpha \left(t\right)=\frac{H_{t}}{\Delta H_{\mathrm{total}}}=\frac{1}{\Delta H_{\mathrm{total}}}\cdot \int_{0}^{t}q\left(t\right)dt$$

Avrami theory was used to describe the kinetic process of polymer crystallization [23]. However, the phase change could also be described clearly with the theory. The UV curing process of the resin can be defined as the change from the liquid phase to the solid phase. In this case, it is possible to predict the UV curing process using the Avrami theory [24,25] and the equation is shown below.

(2) $$\alpha \left(t\right)=1-exp\left(-K\cdot t^{n}\right)$$

where *α* represents the curing degree, K represents the reaction speed constant, *t* represents the reaction time and *n* represents the reaction order.

### 2.3. Parameter Selection

#### 2.3.1. UV Light Intensity

**Figure 4:**
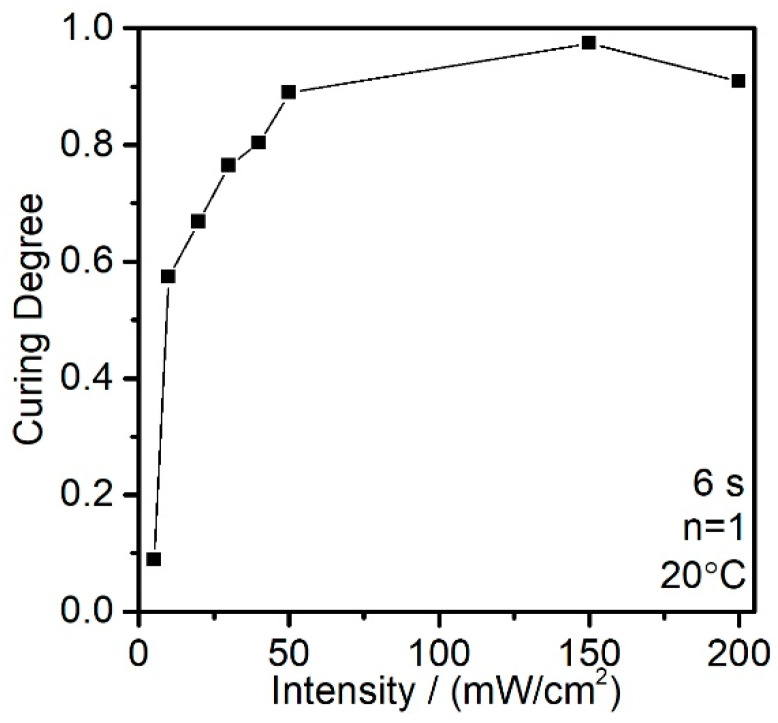
The pretest curing degree with different intensities.

Light intensity is one of the key parameters in the experiments, the level is set based on reference and the technical spec of the equipment [26,27,28]. Several energy levels, 50, 100, 200 mW/cm^2^, were tested as a pretest. The results are shown in Figure 4, which indicates that a relatively high level of curing degree has already been reached at 50 mW/cm^2^ during the process. The higher light intensity will make the results difficult to identify the differences between each parameter. In this case, the range of 5 to 50 mW/cm^2^ was selected to find out the trend of curing degree during the curing process.

#### 2.3.2. Exposure Time

The exposure time was used to estimate the minimum scanning time during the curing process. The curing process is non-linear because the gelation of resin limits the movement of the molecules and the gradually increased *T*_g_ also reduces the reaction rate after a certain curing degree [29,30]. Since the radical process has a fast reaction rate, the curing process could finish in just a few seconds. According to the limitation of the light source shutter speed, the exposure time is designed to start from 1.2 s.

## 3. Results and Discussion

### 3.1. Effects of UV Light Intensity on the Enthalpy and Curing Degree

**Figure 5:**
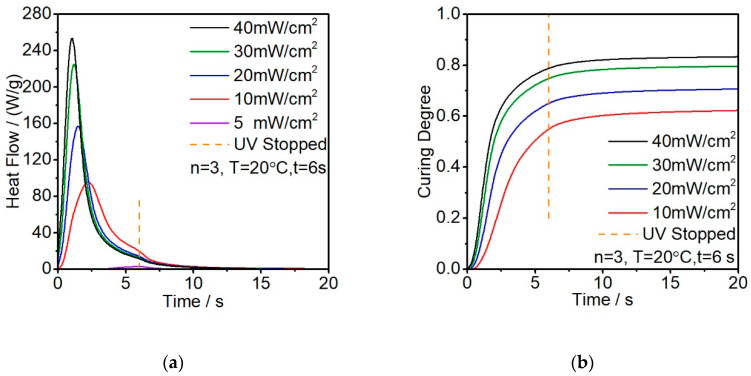
(**a**) Enthalpy analysis with different light intensities; (**b**) calculated curing degree in function with the light intensities.

The influence of the UV light intensity on the photopolymerization process was studied by setting the exposure energy to five different levels. The energy was changed by adjusting the filter between the light guide and the samples. Then, 5, 10, 20, 30, 40 mW/cm^2^ were selected based on the pretests. Figure 5a shows the first irradiation measurement. The results show that the exothermic effects measured by photo-DSC for photopolymerization conducted at 20 °C using various UV light intensities. As the light intensity increases, the amount of exothermic heat tends to increase. The time that the resin reaches the exothermic peak represents the earlier starting point and the higher reaction speed, 40 mW/cm^2^ is about 2.5 s earlier compared to that of 10 mW/cm^2^ and the slope of the curves indicates the difference in the curing rate.

Figure 5b shows the integral curves of the exothermic peaks of Figure 5a. It shows that the sharp reaction rate rises as the light intensity increases. The higher light intensity not only shows stronger exothermic reaction during the curing but also reduces the reaction induction time. This effect might due to the fact that the critical energy consumption exceeds the critical energy level faster. The 40 mW/cm^2^ intensity releases 260 W/g during the first UV light exposure while the 10 mW/cm^2^ intensity releases only 90 W/g. The 5 mW/cm^2^, on the contrary, shows a very low reaction activity that is almost regarded as unreacted during the first exposure.

The slope of the curing degree represents the curing rate. Since the resin volume is fixed, higher light intensity also makes the curing rate decrease in advance compared to the lower light intensity. This phenomenon is because when the resin already cured to a critical point that the molecular could no longer move freely and the *T*_g_ also gradually increased during the curing process until it reached a temperature higher than the environment temperature. From Figure 5b, we could find out that the resin almost reaches the plateau after exposing for 6s, while with 5 mW/cm^2^ the resin remains at a low curing degree. The measured and calculated results of light intensity are shown in Table 1.

### 3.2. Effect of UV Exposure Time on the Enthalpy and Curing Degree

UV exposure time was normally treated as a fixed parameter to observe the trend of the UV light intensity. However, since the pretest showed that the curing degree is not simply linear relative to the enthalpy, it is necessary to investigate the effect of exposure time on the curing behavior.

**Figure 6:**
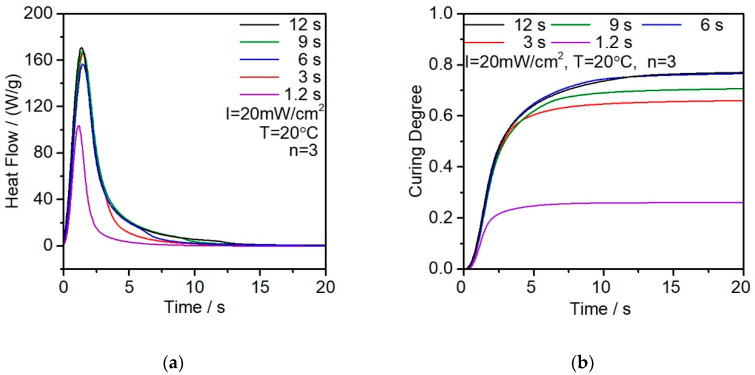
(**a**) Enthalpy analysis with different exposure times; (**b**) calculated curing degree in function with the exposure time.

The results, as shown in Figure 6a, show that longer exposure time will slightly increase the reaction heat. Since the concentration of photoinitiator and the light intensity in these experiments are fixed, the reaction rate shows the same tendency that the heat flow reaches the peak at almost the same time at 2 s. The exposure time of 1.2 s is relatively too short that the heat flow is significantly lower than that of the other parameters.

The curing degree reflects the close behavior of heat flow with different exposure times. Except for the group with 1.2 s which the curing degree only reached 25% because of the short time exposure. The other four exposure time groups all reached at least 70% of the curing degree. As shown in Figure 6b, when exposure time exceeds 3 s, there is no significant improvement in the curing degree of the resin. In this case, it can be concluded that for this fixed amount of resin, the exposure time is considered as enough for 3 s under 20 mW/cm^2^. The measured and calculated results of exposure are shown in Table 2.

**Figure 7:**
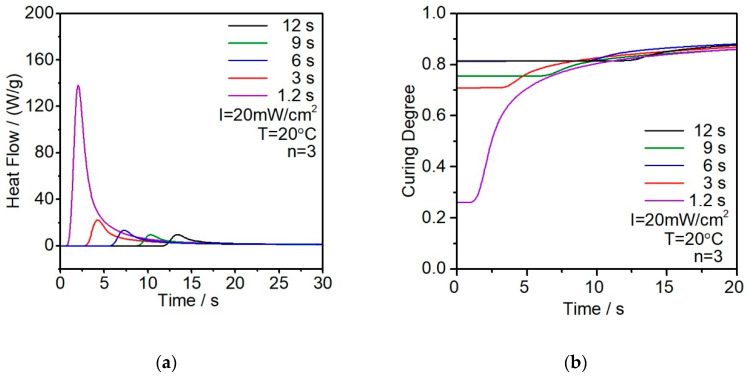
Thermal analysis during the second curing stage (**a**) Enthalpy analysis with different exposure times; (**b**) calculated curing degree in function with the exposure time.

The second exposure also reinforces the result as shown in Figure 7. Since the first exposure 1.2 s resin has relatively limited exposure time, the second exposure shows a sharp enthalpy increasing. The other four curves do not show any significant difference as they already reached at least 70% curing degree at the first exposure. Since all the light intensities are fixed at 20 mW/cm^2^, the second exposure accelerates the process for all the samples to reach almost the same final curing degree.

### 3.3. UV Curing Kinetic Model Parameters Analysis

The reaction kinetics of the photopolymer was studied by using the Johnson–Mehl–Avrami model presented in Equation (2). The Avrami model is usually used for isothermal phase transfer. To accurately present the UV curing kinetics, reaction speed constant K and reaction order *n* was replaced by undetermined coefficient *a* and *b* which was presented in Equation (3). In Equations (4) and (5), *a* and *b* are the first-order polynomials that are related to light intensity *I*, while *c*, *d*, *e* and *f* are undetermined constants.

(3) $$\alpha \left(t\right)=1-exp\left[-a\cdot t^{b}\right]$$

(4) a(I)=c·I+d

(5) b(I)=e·I+f

**Figure 8:**
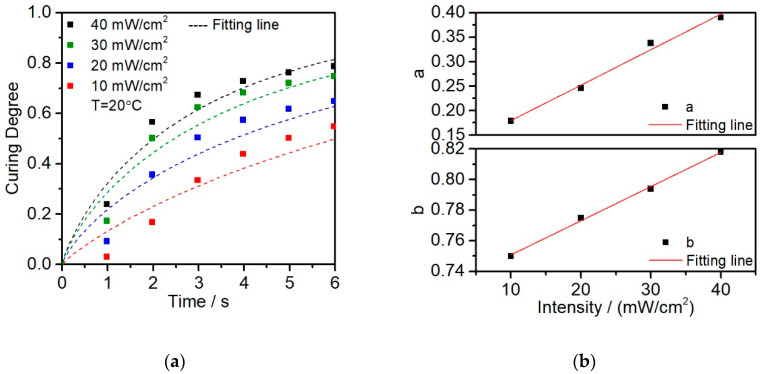
(**a**) Curing degree with different light intensity and related fitting curves by modified Avrami model; (**b**) experiment points and fitting line of undetermined coefficients *a* and *b*.

To find out the undetermined coefficient kinetic parameters *a* and *b*, five different light intensity isothermal photo-DSC experiments were carried out for the resin. Except for 5 mW/cm^2^, which curing degree is too low compared to the others, the other intensities show the high determination of the fitted results. The Avrami model was used to fit the photo-DSC experimental data, the coefficients of determination *R*^2^ related to the four different light intensities are 0.975, 0.957, 0.960, 0.950, respectively. As shown in Figure 8a. All the fitted kinetic parameters are listed in Table 3.

A clear linear dependence of parameters *a* and *b* can be observed and fitting to get the linear polynomial equation as Equations (4) and (5). In this equation, *a* and *b* are the parameters related to light intensity.

To calculate the result of *a* and *b*, Origin was used with fitting linear, and the fitting results are shown in Equations (6) and (7). The fit curve was shown in Figure 8b with *R*^2^ > 0.99.

(6) $$a=0.107+\left(7.25*10^{-3}\right)\cdot I$$

(7) $$b=0.728+\left(2.23*10^{-3}\right)\cdot I$$

where *I* is the light intensity. The linear equations of parameter *a* and *b* were substituted into Equation (3). According to the linear fitting results, the undetermined coefficient *a* and *b* from the polynomial equation can be fit as follows:

(8) $$\alpha \left(t,I\right)=1-exp\left\{-\left[0.107+\left(7.25*10^{-3}\right)I\right]*t^{\left[0.728+\left(2.23*10^{-3}\right)I\right]}\right\}$$

Curing degree α(t,I) is a dependent variable that related UV light intensity *I* and exposure time *t*. With this equation, the curing degree of this resin could be estimated during the curing process by changing time and light intensity.

## 4. Conclusions and Outlook

In this work, thermal properties and curing kinetics were characterized for the customized photosensitive resin. Two important parameters: UV light intensity (5–40 mW/cm^2^) and light exposure time (1.2–12 s) were investigated to find out the influences on the curing degree. The higher light intensity leads to a higher curing rate and curing degree. The critical energy level was found to initiate the reaction in the time frame of UV-assisted direct writing technology. And the exposure time on the curing degree has a plateau that remains relatively unaffected by a short increase of the exposure time. However, when the exposure time is long enough, it accumulates energy and will eventually reach the cured stage. Kinetic analysis was selected with modified Avrami theory which used to describe the phase changing process. The model was established to help to estimate the acrylate resin curing degree with different light intensity and exposure time. With the relationship between the curing degree, light intensity and exposure time, this equation could help to optimize the processing parameters for the UV-assist direct writing before the experiment.

For future research, it is interesting to discover the influence of material overcuring, deformation and shrinkage, viscosity and the critical curing degree, which will be essential to the printing process. To better understand the changing during curing, the combination of rheological and real-time IR properties of the resin system, filled resin system curing behavior will also wait to be discussed to analyze the influence of the processing parameters on material properties.

## Figures and Tables

**Table 1:** Properties analysis results for different light intensities.

Light Intensity (mW/cm^2^)	ΔH (J/g)	$\Delta H_{\mathrm{total}}$ (J/g)	Peak Time (s)	Curing Degree (%)
5	8.01	259.62	6	3.1
10	242.3	541.76	2.28	67.09
20	275.47	548.29	1.48	75.33
30	475.67	568.91	1.18	83.63
40	424.21	571.54	1.08	87.07

**Table 2:** Thermal properties analysis results for different exposure times.

Exposure Time (s)	ΔH (J/g)	$\Delta H_{\mathrm{total}}$ (J/g)	Peak Time (s)	Curing Degree (%)
1.2	182	568.38	1.18	26.85
3	398.15	560.79	1.48	70.98
6	275.47	548.29	1.48	75.33
9	462.82	568.28	1.48	81.44
12	461.08	564.1	1.38	81.74

**Table 3:** Parameters used in the modified Avrami model Equation (3).

	10 mW/cm^2^	20 mW/cm^2^	30 mW/cm^2^	40 mW/cm^2^
*a*	0.179	0.246	0.338	0.39
*b*	0.878	0.775	0.794	0.818
